# Inflammation Disrupts the LDL Receptor Pathway and Accelerates the Progression of Vascular Calcification in ESRD Patients

**DOI:** 10.1371/journal.pone.0047217

**Published:** 2012-10-24

**Authors:** Jing Liu, Kun Ling Ma, Min Gao, Chang Xian Wang, Jie Ni, Yang Zhang, Xiao Liang Zhang, Hong Liu, Yan Li Wang, Bi Cheng Liu

**Affiliations:** 1 Institute of Nephrology, Zhong Da Hospital, Southeast University School of Medicine, Nanjing City, Jiangsu Province, People’s Republic of China; 2 Department of Infection Management, Zhong Da Hospital, Southeast University School of Medicine, Nanjing City, Jiangsu Province, People’s Republic of China; University of Milan, Italy

## Abstract

**Background:**

Chronic inflammation plays a crucial role in the progression of vascular calcification (VC). This study was designed to investigate whether the low-density lipoprotein receptor (LDLr) pathway is involved in the progression of VC in patients with end-stage renal disease (ESRD) during inflammation.

**Methods and Results:**

Twenty-eight ESRD patients were divided into control and inflamed groups according to plasma C-reactive protein (CRP) level. Surgically removed tissues from the radial arteries of patients receiving arteriovenostomy were used in the experiments. The expression of tumour necrosis factor-α (TNF-α) and monocyte chemotactic protein-1 (MCP-1) of the radial artery were increased in the inflamed group. Hematoxylin-eosin and alizarin red S staining revealed parallel increases in foam cell formation and calcium deposit formation in continuous cross-sections of radial arteries in the inflamed group compared to the control, which were closely correlated with increased LDLr, sterol regulatory element binding protein-2 (SREBP-2), bone morphogenetic proteins-2 (BMP-2), and collagen I protein expression, as shown by immunohistochemical and immunofluorescent staining. Confocal microscopy confirmed that inflammation enhanced the translocation of the SREBP cleavage-activating protein (SCAP)/SREBP-2 complex from the endoplasmic reticulum to the Golgi, thereby activating LDLr gene transcription. Inflammation increased alkaline phosphatase protein expression and reduced α-smooth muscle actin protein expression, contributing to the conversion of the vascular smooth muscle cells in calcified vessels from the fibroblastic to the osteogenic phenotype; osteogenic cells are the main cellular components involved in VC. Further analysis showed that the inflammation-induced disruption of the LDLr pathway was significantly associated with enhanced BMP-2 and collagen I expression.

**Conclusions:**

Inflammation accelerated the progression of VC in ESRD patients by disrupting the LDLr pathway, which may represent a novel mechanism involved in the progression of both VC and atherosclerosis.

## Introduction

Cardiovascular disease (CVD) is the leading cause of morbidity among patients with end-stage renal disease (ESRD), accounting for approximately 50% of deaths and 30% of hospitalisations in this population [1]. Annual CVD mortality is 10–20 fold higher in ESRD patients than in the general population, and this difference is not completely explained by traditional risk factors [2]. Recently, more attention has been paid to vascular calcification (VC), which induces arterial stiffness, high pulse pressure, and cardiac valve dysfunction, contributing to ventricular hypertrophy and heart failure [3], [4]. Thus, VC results in an increased risk of CVD mortality, especially in ESRD patients, regardless of maintenance hemodialysis (HD) treatment status.

Vascular calcification is a complicated pathological process that develops primarily within the intimal and medial layers of the artery. Arterial intimal calcification (AIC) is an advanced form of atherosclerosis (AS), driven by cellular necrosis, inflammation, and lipid deposition manifested in a patchy, discontinuous course along the artery. Specific risk factors for AIC in uraemia patients include hyperphosphatemia, hypoalbuminemia, excessive calcium intake, and HD duration. Arterial medial calcification (AMC) is observed in the elastic lamella of the medial layer of the arteries. AMC is closely associated with HD duration even in patients with no CVD history at HD therapy onset. AMC is an active process that involves the transformation of medial vascular smooth muscle cells (VSMCs) from a fibroblastic to an osteogenic phenotype. Normally, VSMCs have a contractile phenotype and constitutively express proteins that inhibit mineralisation. In response to various stimuli, however, VSMCs express and/or release several key regulators of bone formation and bone structural associated proteins, such as bone morphogenetic protein-2 (BMP-2), alkaline phosphatase (ALP), and collagen I. In contrast, the expression of proteins such as α-smooth muscle cell (α-SMA) and collagen IV is reduced, ultimately transforming VSMCs into osteoblast-like cells [5], [6]. However, the precise mechanisms that cause the osteogenic phenotype of VSMCs in calcified vessels are not completely clear.

Chronic systemic inflammation is a common feature in ESRD patients [7], and it may be correlated with the accumulation of pro-inflammatory compounds caused by a markedly decreased glomerular filtration rate (GFR) [8]. Other causes, including malnutrition, metabolic acidosis, hyperparathyroidism, the accumulation of advanced oxidation protein products and asymmetric dimethyl arginine, contribute to the release of inflammatory cytokines [9]. Inflammation accelerates the progression of AS and VC [10], [11], which has been identified as an independent risk factor for the morbidity and mortality of CVD in ESRD patients [12].

It is well known that the low-density lipoprotein receptor (LDLr) pathway is a feedback system with important roles in regulating plasma and intracellular cholesterol homeostasis, and it is mainly modulated by the concentration of intracellular cholesterol and the interaction between sterol regulatory element binding protein (SREBP) and SREBP cleavage-activating protein (SCAP). Cholesterol deficiency enhances the translocation of SCAP from the endoplasmic reticulum (ER) to the Golgi, where it cleaves SREBP, thus increasing LDLr gene expression.

Our previous studies demonstrated that inflammation accelerated the progression of AS by disrupting LDLr feedback regulation [13], [14]. The present study was performed to evaluate whether the inflammation exacerbates the progression of VC in ESRD patients and explore the underlying mechanisms.

## Materials and Methods

### Ethics Statement

All studies were approved by the Ethical Committee of Southeast University. Each patient provided written informed consent to the use of their tissues for research purposes.

### Patient Selection and Clinical Data

We studied 28 ESRD patients from Zhong Da Hospital, Southeast University between January 2010 and May 2011. Patients with ESRD who were to undergo arteriovenostomy before hemodialysis were included in the study. Patients with acute infection, cancer, and/or chronic active hepatitis were excluded. The included patients were divided into two groups based on plasma C-reactive protein (CRP) levels: control (CRP<3.0 mg/l, n = 14) and inflamed (CRP> = 3.0 mg/l, n = 14) group. Inflamed group was defined as the patients with persistent increased plasma levels of CRP checked at the start and the second week after hospitalization. The patients were comprehensively monitored by the symptoms, signs, and serum indexes, in order to detect timely any confounding condition which may potentially affect serum CRP level.

### Clinical Biochemical Tests

Blood samples were assayed to determine erythrocyte sedimentation rate (ESR), CRP, red blood cells (RBC), haemoglobin (Hb), total protein (TP), albumin (ALB), glucose (GLU), triglyceride (TG), total cholesterol (TC), low density lipoprotein (LDL), high density lipoprotein (HDL), apolipoprotein A1 (Apo A1), Apo B, lipoprotein (a), calcium (Ca), phosphate (P), and intact parathyroid hormone (iPTH) using an automatic biochemistry analyser at the clinical chemistry centre of the hospital.

### Tissue Processing

Tissues from the radial artery were taken during radial-cephalic anastomosis surgery. The tissue sections were rinsed with saline and placed in 10% buffered formalin. After treatment, representative sections of the grafts were obtained and embedded in O.C.T. medium or paraffin.

### Hematoxylin-eosin (HE) Staining

The paraffin embedded tissues were sliced and dewaxed. The slices were dyed for 15 minutes with hematoxylin, dipped in 1% hydrochloric alcohol, and then stained with 1% eosin for 3 minutes. The slices were sealed with resinene after they were dehydrated to transparency. The results were observed under light microscope (×200).

### Alizarin red S Staining

The paraffin-embedded tissues were dewaxed and hydrated in 70% alcohol. After rinsing rapidly in distilled water, the slices were dyed with alizarin red S solution for one minute. Positive results were shown in an orange-red colour in light microscope (×200).

### Immunohistochemical Staining

Paraffin-embedded sections (4 µm) were subjected to immunohistochemical staining. After deparaffinisation, sections were placed in citrate-buffered solution (pH 6.0) and then heated for antigen retrieval. Endogenous peroxidase was blocked with 3% hydrogen peroxide, and nonspecific antibody binding was blocked with 10% goat serum. Subsequently, sections were incubated with goat, rabbit or mouse anti-human primary antibodies against TNF-α (Santa Cruz, USA), MCP-1 (Santa Cruz, USA), LDLr (Abcam, UK), BMP-2 (Santa Cruz, USA), and collagen I (Abcam, UK) overnight at 4°C, followed by incubation with biotinylated secondary antibodies. Finally, a diaminobenzidine tetrahydrochloride substrate was used to develop the reaction. The results were observed under a light microscope (×200). Semi-quantitative analysis was performed by the software of Image-Pro Plus version 5.0.

### Immunofluorescent Staining

The frozen sections were fixed with 4% formalin solution and then blocked with 5% bovine serum albumin (BSA). Subsequently, the sections were incubated with rabbit, mouse, or goat anti-human primary antibodies against SCAP (Abcam, UK), α-SMA (Abcam, UK), Golgi (Invitrogen, USA), and ALP (Santa Cruz, USA), followed by goat anti-rabbit Fluor 488, donkey anti-rabbit Fluor 594, goat anti-mouse Fluor 594, and donkey anti-goat Fluor 488 secondary ﬂuorescent antibodies (Invitrogen, USA), respectively. After washing, the slides were examined by laser confocal microscopy (×100). Semi-quantitative analysis was performed by the software of Image-Pro Plus version 5.0.

### Statistical Analysis

All the data were expressed as the mean ± standard deviation (SD) and were analysed with SPSS 13.0. Continuous variables were compared between the two groups with the independent-sample *t* test (where appropriate), and correlations between variables were analysed by Spearman’s R coefficient. A difference was considered significant if the *P* value was less than 0.05.

## Results

### Basic Clinical Data of the Patients in the Two Groups

As shown in Table 1, there were no differences in age, body weight, ESR, RBC, Hb, TP, ALB, lipid profiles, Ca, P, Ca×P, or iPTH (P>0.05) between the inflamed group and the control group.

**Table 1:** Basic clinical and biochemical data for the patients.

Parameters	control (n = 14)	inflamed group (n = 14)
Weight(Kg)	63.71±9.82	63.54±12.47
Age (ys)	49.07±16.34	57.71±17.32
RBC(10^12^/L)	2.57±0.57	2.72±0.63
Hb(g/L)	76.89±17.39	78.67±18.29
TP(g/L)	57.07±10.51	57.36±9.6
ALB(g/L)	28.14±6.20	28.36±4.89
GLU (mmol/L)	5.34±2.46	5.52±1.92
TG (mmol/L)	1.33±1.06	1.18±0.64
T-CHO (mmol/L)	4.81±1.26	4.51±1.00
LDL (mmol/L)	3.09±1.09	2.83±0.87
HDL(mmol/L)	1.21±0.28	1.08±0.37
ApoA1 (mmol/L)	1.11±0.23	0.97±0.29
ApoB (mmol/L)	0.89±0.30	0.81±0.21
LP(a) (mmol/L)	395.43±204.79	366.43±203.45
Ca (mmol/L)	2.00±0.24	2.01±0.27
P (mmol/L)	1.89±0.31	1.83±0.47
Ca*P (mmol/L)^2^	3.75±0.55	3.57±0.54
iPTH (pg/mL)	255.84±168.57	205.05±133.97

There was no difference compared every index in the inflamed group with that in the control, *P*>0.05.

### Local Upregulation of Inflammation in the Artery was Consistent with Plasma CRP Level

**Figure 1:**
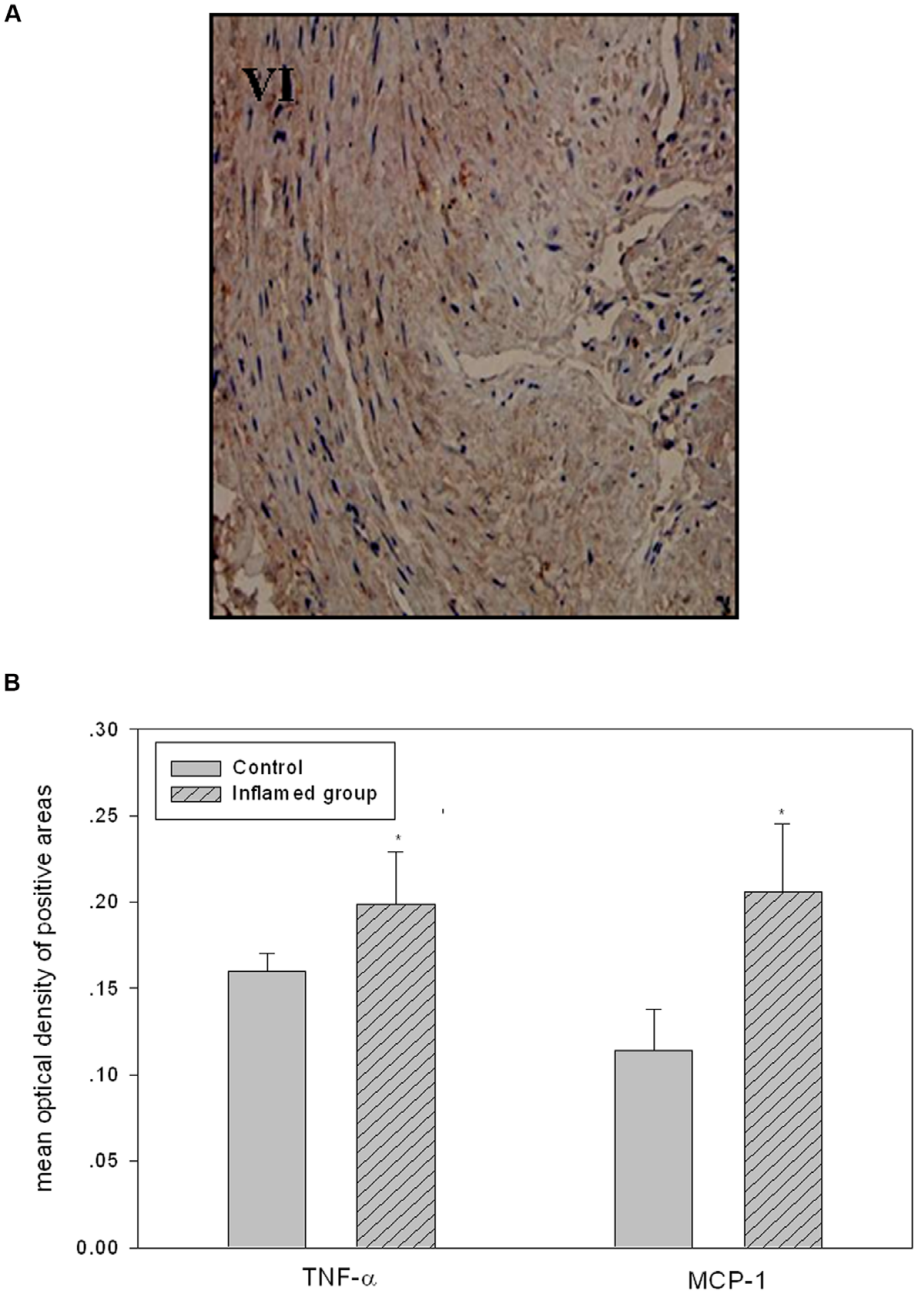
Locally upregulated inflammation in the artery was consistent with the plasma CRP level. The local inflammation status in the radial artery was examined by immunohistochemical staining. The positive areas were stained brown in cross-sections of radial arteries (Fig. 1, I–VI, original magnification ×200). The values of semiquantitative analysis for the positive areas were expressed as the mean ±SD from five patients in each group (n = 14). * *P*<0.05 *vs* control (Fig. 1B).

Using immunohistochemical staining, we demonstrated that the expression of TNF-α and MCP-1 were increased in the radial artery in the inflamed group, which indicated that the local inflammation in the artery was upregulated, consistent with the observation of systemic inflammation stress (Fig. 1A, Fig. 1B).

### Inflammation Induced Foam Cell Formation and Calcified Plaque Deposition of Radial Arteries

**Figure 2:**
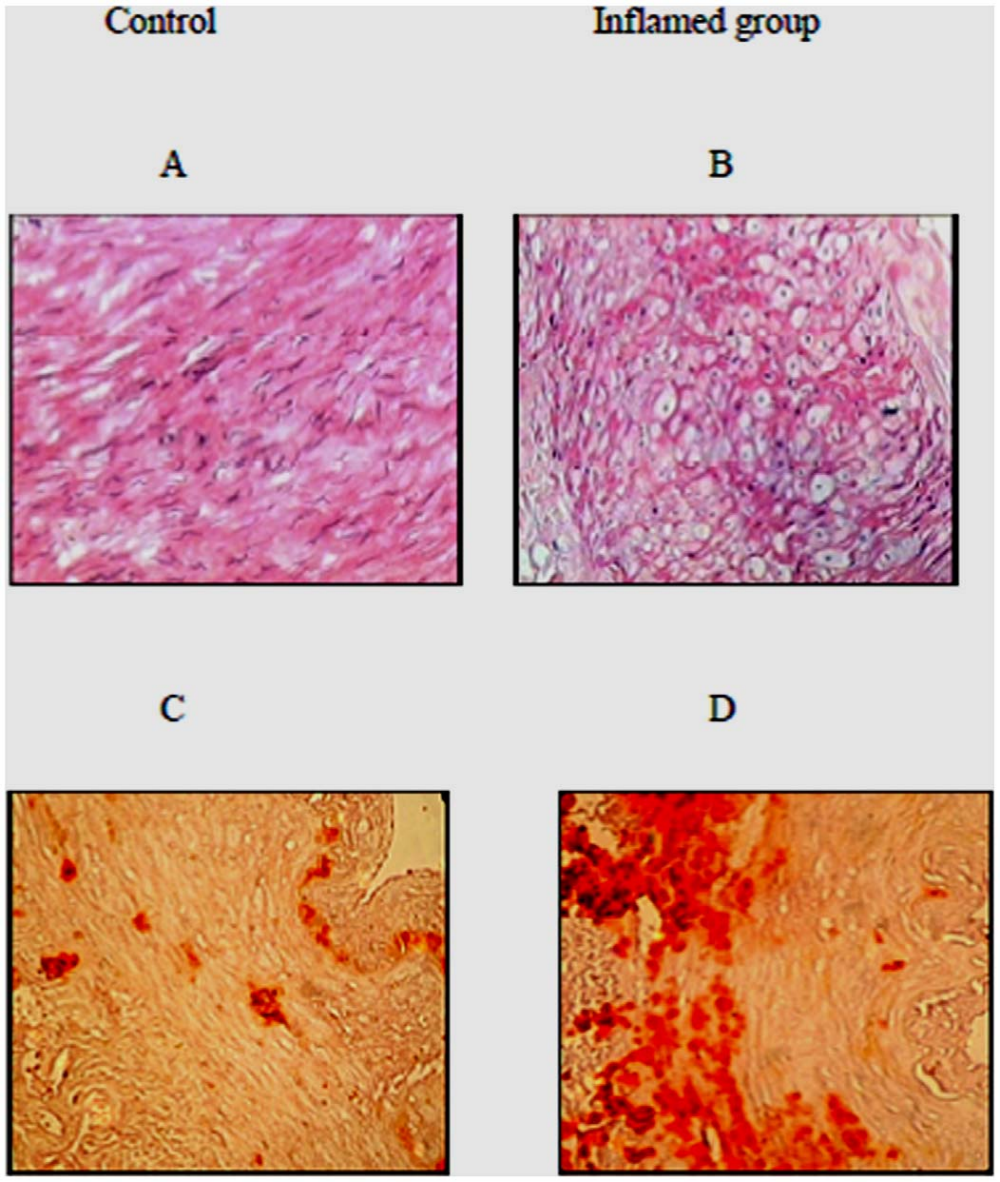
Inflammation induced foam cell formation and calcified plaque deposition in the radial arteries. The lipid accumulation in the radial arteries was checked by hematoxylin-eosin staining (Fig. 2A and Fig. 2B, original magnification ×200) Calcification was examined by alizarin red S staining, and calcium deposits were stained orange-red (Fig. 2C and Fig. 2D, original magnification ×200).

HE staining showed that there was significant foam cell formation in the radial arteries of the inflamed group compared to the control (Fig. 2A, Fig. 2B). Interestingly, there was a parallel increase in calcified plaque deposition in the radial arteries of the inflamed group compared to controls, as evaluated by alizarin red S staining, suggesting that a common mechanism could be involved in both AS and VC (Fig. 2C, Fig. 2D).

### Inflammation Disrupted the Feedback Regulation of the LDL Receptor

**Figure 3:**
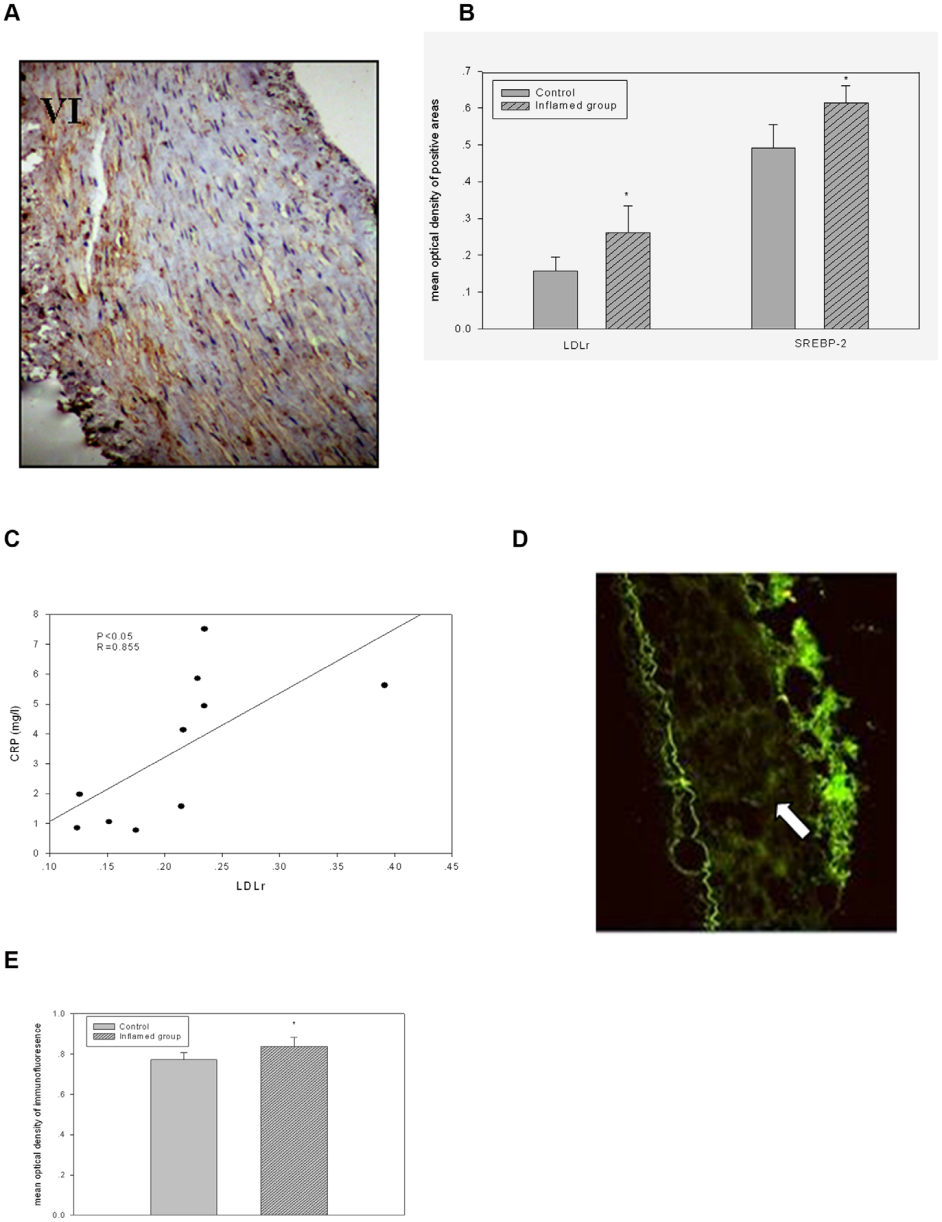
Inflammation disrupted the feedback regulation of the LDL receptor. LDLr and SREBP-2 protein expression were measured by immunohistochemical staining. The positive areas were stained brown in radial artery cross sections (Fig. 3A I–VI, original magnification ×200). The values of semiquantitative analysis for the positive areas are expressed as the mean ±SD from five patients at each group (n = 14). * *P*<0.05 *vs* control (Fig. 3B). Correlation analysis of plasma CRP level with LDLr expression (Fig. 3C). The translocation of SCAP from the ER to Golgi was evaluated by immunofluorescent staining. SCAP and Golgi are stained in green and red, respectively. The colocalisation of SCAP with Golgi was evaluated by laser confocal microscopy (Fig. 3D, arrow indicates colocalisation, original magnification ×100). Overlay areas were quantified and expressed as the mean ± SD from five patients at each group (n = 14). * *P*<0.05 *vs* control (Fig. 3E).

To explore the potential mechanisms underlying this phenomenon, we evaluated the effects of inflammation on the protein expression of LDLr and SREBP-2 by immunohistochemical staining in radial arteries. Inflammation significantly increased LDLr and SREBP-2 protein expression (Fig. 3A I–VI, Fig. 3B). Moreover, the plasma CRP level was positively correlated with the expression of the LDLr protein (Fig. 3C). Therefore, we further investigated the effect of inflammation on the translocation of SCAP escorting SREBP-2 from the ER to the Golgi in the radial arteries. Confocal microscopy showed that inflammation significantly increased SCAP translocation from the ER to the Golgi, thereby activating LDLr gene transcription (Fig. 3D, Fig. 3E.).

### Inflammation Accelerated VC by Contributing to VSMC Conversion from the Fibroblastic to the Osteogenic Phenotype

**Figure 4:**
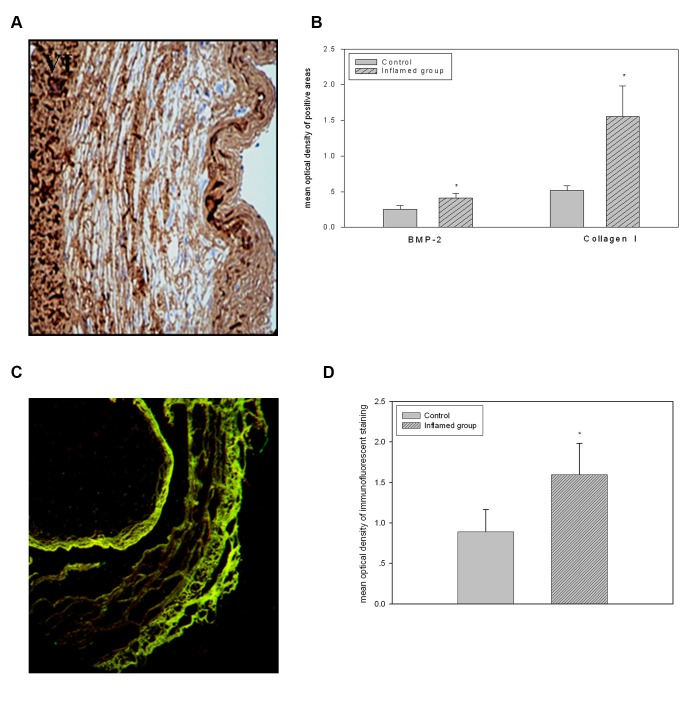
Inflammation accelerated the VC by contributing to the phenotype conversion of VSMC from the fibroblastic to the osteogenic. The protein expressions of BMP-2 and Collagen I were checked by immunohistochemical staining. The positive areas were stained as brown in cross-sections of radial arteries (Fig. 4A I–VI, original magnification×200). The values of semiquantitative analysis for the positive areas were expressed as the mean ±SD from five patients at each group (n = 14). * *P*<0.05 *vs* control (Fig. 4B). The co-expression of the ALP and α-SMA proteins was checked by immunofluorescent double staining. ALP and α-SMA are stained with green and red fluorescence, respectively (Fig. 4C, original magnification ×100). The ratio of green to red in the overlay areas were quantified and expressed as the mean±SD from five patients in each group (n = 14). * *P*<0.05 *vs* control (Fig. 4D).

To investigate the possible mechanisms of VC in the context of inflammation, we evaluated the effects of inflammation on the expression of the bone formation biomarkers BMP-2 and collagen I in the radial arteries during VC progression. As shown by immunohistochemical staining, BMP-2 and collagen I protein expression were significantly increased in the inflamed group compared to the control group (Fig. 4A I–VI, Fig. 4B). It is well known that VSMC is one of the major cellular components involved in the progression of AMC. Therefore, we evaluated the protein expressions of ALP and α-SMA. As shown by immunofluorescent staining, inflammation significantly increased ALP expression and decreased α-SMA expression (Fig. 4C, Fig. 4D). This suggests that inflammation induces VSMC conversion from the fibroblastic to the osteogenic phenotype, thereby accelerating the progression of AMC.

### The Disruption of the LDLr Pathway was Closely Associated with AS and VC of the Radial Arteries

**Figure 5:**
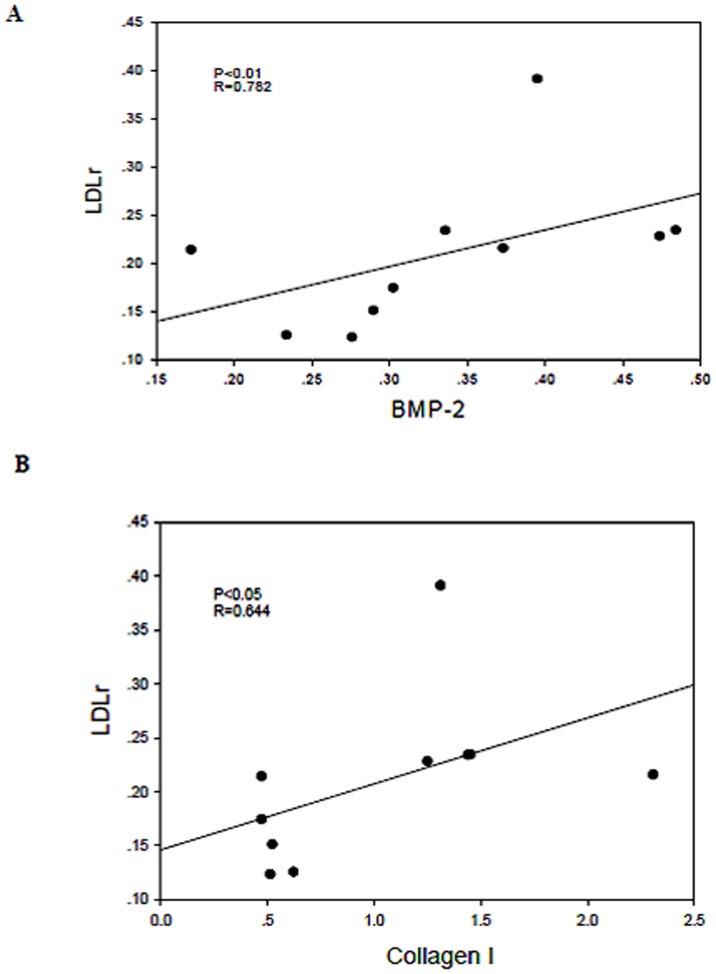
The disruption of the LDLr pathway was closely associated with VC of the radial arteries. Correlation analysis demonstrated that LDLr protein expression was positively associated with BMP-2 (Fig. 5A, R = 0.782, P<0.01) and collagen I (Fig. 5B, R = 0.644, P<0.05) expression.

Correlation analysis demonstrated that LDLr protein expression was positively associated with BMP-2 and collagen I protein expression (Fig. 5A and Fig. 5B). These findings, in combination with those of our previous studies, suggest that the disruption of the LDLr pathway under inflammatory stress may be closely associated with the progression of AS and VC.

## Discussion

Dyslipidemia and chronic inflammation are common complications in chronic kidney disease (CKD), especially ESRD. It has been reported that dyslipidemia and inflammation act together as “partners in crime” to accelerate the progression of AS and vascular calcification in HD patients. Our previous *in vivo* and *in vitro* studies showed that inflammation induced intracellular lipid accumulation and foam cell formation by disrupting LDLr feedback regulation, exacerbating the progression of AS [13]–[15]. The present study was designed to investigate whether the LDLr pathway was involved in the progression of VC in HD patients under inflammatory stress.

Using immunohistochemical staining, we found that TNF-α and MCP-1 protein expression were increased in the arteries of the inflamed group compared to controls, suggesting that local arterial inflammation was also upregulated in the inflamed group.

By evaluating continuous cross-sections, we demonstrated that there was a significant increase in foam cell formation and calcium deposition in the inflamed group, as shown by hematoxylin eosin and alizarin red S staining, respectively. The parallel pathological changes in AS and VC appeared in the same area of the radial artery, suggesting that a common pathway mediated by inflammation and dyslipidemia may be involved in the progression of both AS and VC.

We found that LDLr protein expression in the radial artery tissues was increased in the inflamed group compared to the control group and that this increase was positively associated with plasma CRP level. Further analysis showed that upregulated LDLr protein expression was mediated by increased SREBP-2 protein expression and enhanced SCAP/SREBP-2 complex translocation. These clinical findings were consistent with our previous *in vivo* and *in vitro* studies, showing that the disruption of the LDLr pathway played crucial roles in the progression of AS.

It is accepted that AMC, the main calcification mechanism in CKD patients, is closely associated with the conversion of VSMCs from fibroblastic to osteogenic phenotypes and the upregulation of osteogenic programs [16]. Although Proudfoot *et al* reported that acetylated LDL stimulates human VSMC calcification by promoting osteoblastic differentiation, little is known about the roles of LDLr feedback regulation in modulating the phenotype conversion of VSMCs in calcified vessels in the context of inflammation. To investigate the possible link between the disruption of the LDLr pathway and VC, we further evaluated the effects of inflammation on AMC in the tissues of the radial artery. Using tissues removed from the radial arteries of HD patients, we demonstrated that inflammation significantly increased the expression of the bone formation biomarker proteins BMP-2 and collagen I during VC progression. Immunofluorescent staining showed that inflammation significantly increased ALP expression and decreased α-SMA expression. The correlation analysis further showed that LDLr protein expression was positively associated with BMP-2 and collagen I protein expression. These observations suggest that inflammation accelerates the progression of AMC by disrupting the LDLr pathway, which is closely associated with the induction of VSMC phenotype conversion. Recently, using LDL receptor deficient mice, Geng Y *et al* [17] demonstrated that up-regulation of cholesterol metabolism is essential for matrix mineralization by vascular cells, which were further confirmed by our findings.

In summary, our findings demonstrate for the first time that inflammation accelerates the progression of VC in HD patients by disrupting the LDLr pathway. This may be a novel mechanism involved in the progression of both VC and AS.

### Limitations

The main limitation was low number of patients and small size of samples acquired from radial arteries in two groups, which may limit providing more evidence with the evaluation of VC. CRP, as a biomarker in this study for the evaluation of inflammatory status in ESRD patients, should be understood objectively. Although it has been widely accepted as a valuable and well recognized index of inflammation, some studies recently have reported that CRP partly depends upon commonly unmeasured factors (e. g. genetic traits) [18], [19]. In addition, as each patient had different conditions, such as age, complications, treatments, serum levels of phosphate, calcium, and intact parathyroid hormone, the data could be not well-controlled.
