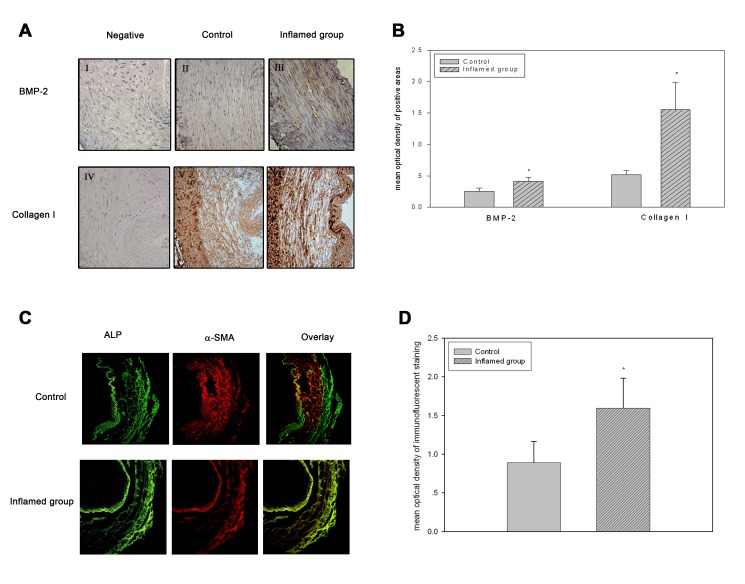# Correction: Inflammation Disrupts the LDL Receptor Pathway and Accelerates the Progression of Vascular Calcification in ESRD Patients

**DOI:** 10.1371/annotation/6d657d25-6835-40ee-9751-3f8fbc40e2b8

**Published:** 2013-08-20

**Authors:** Jing Liu, Kun Ling Ma, Min Gao, Chang Xian Wang, Jie Ni, Yang Zhang, Xiao Liang Zhang, Hong Liu, Yan Li Wang, Bi Cheng Liu

Figures 1, 3, and 4 were incomplete. Complete versions of these figures can be found below:

Figure 1: 

**Figure:**
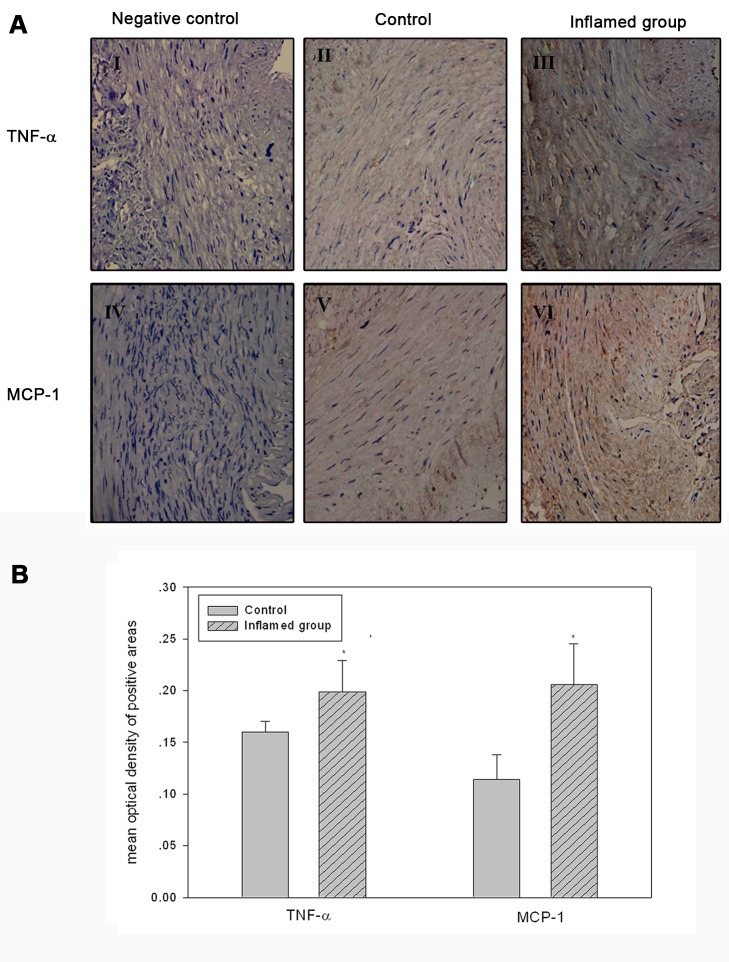


Figure 3: 

**Figure:**
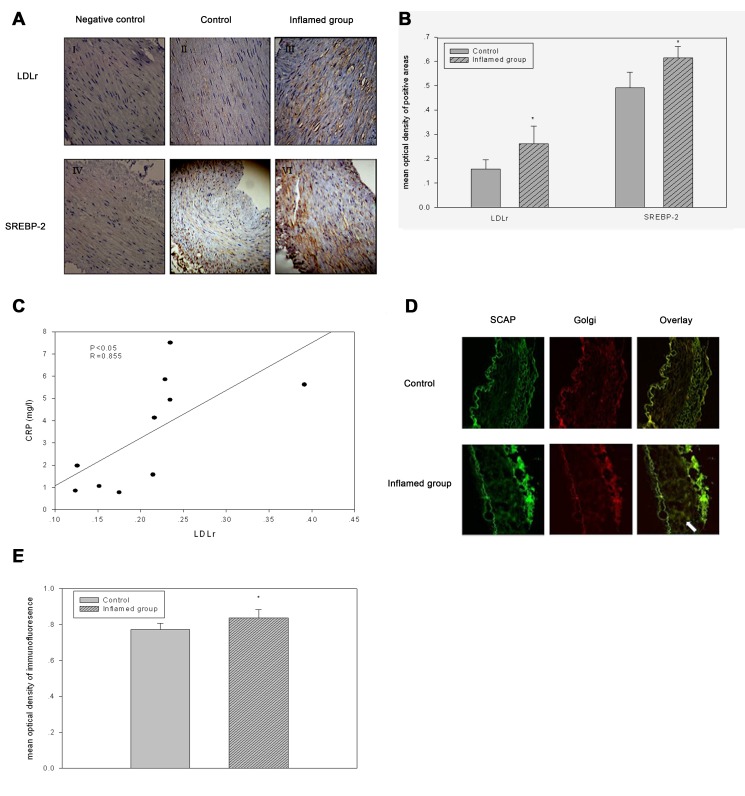


Figure 4: 

**Figure:**